# Out of Sight but Not out of Mind: Alternative Means of Communication in Plants

**DOI:** 10.1371/journal.pone.0037382

**Published:** 2012-05-22

**Authors:** Monica Gagliano, Michael Renton, Nili Duvdevani, Matthew Timmins, Stefano Mancuso

**Affiliations:** 1 Centre for Evolutionary Biology, School of Animal Biology, University of Western Australia, Crawley, Australia; 2 Centre for Microscopy, Characterisation and Analysis, University of Western Australia, Crawley Australia; 3 School of Plant Biology, University of Western Australia, Crawley, Australia; 4 CSIRO Ecosystem Sciences, Floreat, Australia; 5 Metabolomics Australia, Plant Energy Biology, Autralian Research Center (ARC) Centre of Excellence, University of Western Australia, Crawley, Australia; 6 International Laboratory of Plant Neurobiology, Department of Plant, Soil and Environmental Science, University of Firenze, Sesto, Italy; University of Tartu, Estonia

## Abstract

Current knowledge suggests that the mechanisms by which plants communicate information take numerous forms. Previous studies have focussed their attention on communication via chemicals, contact and light; other methods of interaction between plants have remained speculative. In this study we tested the ability of young chilli plants to sense their neighbours and identify their relatives using alternative mechanism(s) to recognised plant communication pathways. We found that the presence of a neighbouring plant had a significant influence on seed germination even when all known sources of communication signals were blocked. Furthermore, despite the signalling restriction, seedlings allocated energy to their stem and root systems differently depending on the identity of the neighbour. These results provide clear experimental evidence for the existence of communication channels between plants beyond those that have been recognized and studied thus far.

## Introduction

Communication is ubiquitous in nature and is arguably one of the most studied topics in the behavioural sciences. While the search for a rigorous and comprehensive definition of communication has been and still is at the heart of much debate [1], [2], the basic phenomenon involves the transfer of some kind of information from one individual to another. Historically, the study of communication processes has primarily focused on animals, probably because their signal-mediated interactions often involve loud and bold displays and eye-catching movements of distinctive body parts, which have clearly succeeded in attracting our attention. On the other hand, the notion of communication in plants has long been regarded as a controversial fringe idea, which has only recently begun to attract more widespread attention [3]–[6]. Yet, plants have now proven to be highly sensitive organisms that interact and facilitate each other by actively acquiring information from their environment. Indeed, research findings over the last decades have demonstrated that plants process and evaluate information about their neighbours both above [7] and below ground [8]–[10], as well as about the resources available in their surroundings, and modify their behaviour accordingly [11]–[12]. For example, plants use information to recognize and even prevent costly competitive interactions with relatives by favouring them over strangers [13]–[14], and hence facilitating kin selection processes such as cooperation and altruism, similar to what is seen in animal social systems.

Our current knowledge suggests that the mechanisms by which plants communicate all this information are complex and take a number of forms. The most recent literature is replete with examples that show how plants communicate through the release of chemicals [15], mechanical contact induced by gravity, thigmo stimuli and changes in pressure gradients of various nature [16] and/or the transmission and reflection of different wavelengths of light [17]. For example, plants can warn each other of approaching insect attacks using an extensive vocabulary of chemical molecules, such as herbivore-induced volatile organic compounds (VOCs). In fact, through this airborne plant-plant communication channel, plants are able to respond to cues produced by injured neighbours when they are not yet attacked or damaged themselves, hence allowing for pre-emptive defensive responses [18]–[20]. Similarly, light-mediated perception of neighbouring plants, and particularly putative competitors, may help plants to budget their investment in defensive efforts. For example, plants have evolved specific photoreceptors (e.g. phytochrome B), which allow them to monitor specific changes in the level of far-red (FR) relative to the red (R) component of sunlight [21] and thus perceive the proximity of a future competitor. Because plants are unable to simultaneously invest their limited resources in growth as well as defence [22], the perception of such spectral changes that signal the advent of increased competition before any actual shortage of resources takes place is clearly beneficial. In response to the presence of competitors, plants can shape their morphology and adjust future growth accordingly.

Plant communication by means of chemicals, contact or light wavelengths is now well recognised, and the study of these types of communication is well under way. We hypothesised that plants also employ other alternative ways of communicating, based on sound or magnetic waves for example. Therefore the aim of this study was to look for evidence of such alternative means of communication, by testing whether any interaction between plants still occurs when all communication based on recognised means has been blocked. In particular we asked (1) whether the presence of a neighbouring plant could influence germination rates of seeds when above- and below-ground contact, chemical and light-mediated signals are blocked; and if so, (2) whether such effects on germination and growth differed depending on the identity of the neighbouring plant (i.e. conspecific vs heterospecific).

## Methods

### Model Species

As our model system, we used the seeds of *Capsicum annuum* (Solanaceae), a widespread chilli species originally native to the Americas where it has been domesticated for over 6,000 years [23], which is now cultivated worldwide in its many varieties. The commercially cultivated types of this flowering plant produce large fruits, which are green in colour ripening into red, and have lost their natural mechanisms for seed dispersal [24]. To test whether the presence of a neighbouring plant influenced how chilli seeds germinated and grew, we chose the Florence fennel plant (*Foeniculum vulgare*, Apiaceae). *F. vulgare* was a particularly appropriate heterospecific neighbour for this study, because this species is known to exude chemicals from roots or aerial parts that inhibit growth and even kill its neighbours so is generally grown in seclusion [25]. Hence, we expected the presence of fennel to retard or block germination and/or growth rates of chilli when open contact was possible and to have a progressively smaller negative effect on germination as its signals were partially or totally blocked.

### Experimental Set Up and Procedures

**Figure 1:**
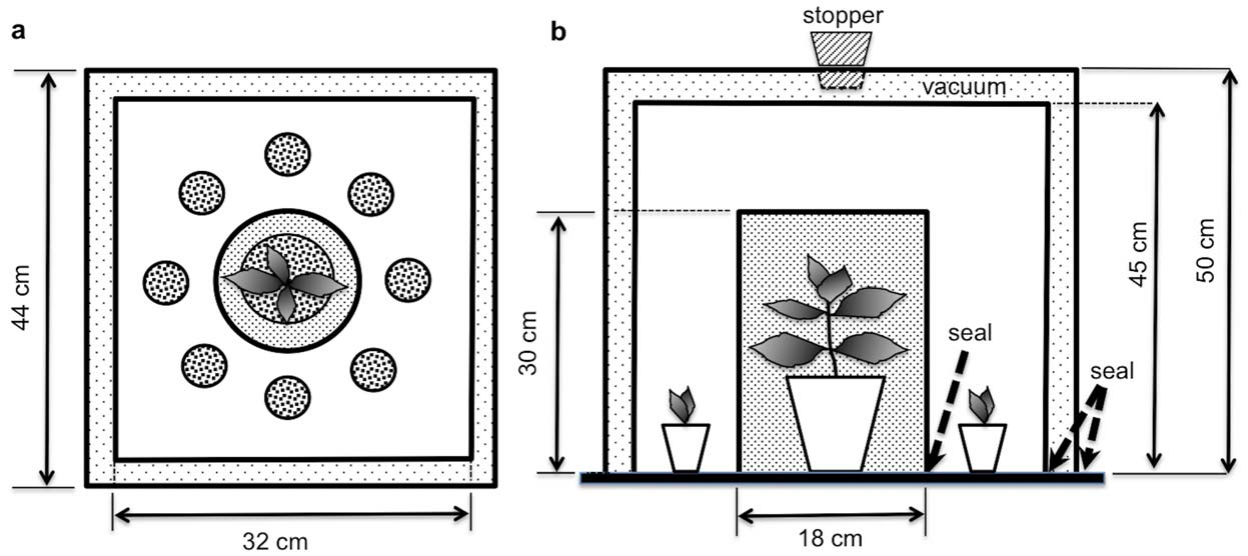
Schematic representation of the custom-designed experimental unit (not in scale). (**a**) The seal at the base of the central cylindrical box ensured that chilli seeds arranged in a circle around the adult plant were chemically isolated from it. (**b**) All seeds and adult plants within a replicate unit were housed within 2 different sized square boxes, one inside the other, with the air in between the two boxes removed using a vacuum pump. The whole experimental unit was custom-made in colourless cast acrylic material (ModenGlas), which transmitted 92% of visible light, but was opaque to ultraviolet and infrared wavelengths.

All experiments were conducted at the Plant Growth Facilities at the University of Western Australia. Experiments were done in a 5.30 m^2^ Controlled Environment Room (CER) fitted with high-intensity discharge lamps. We used custom-designed experimental units (Figure 1), which prevented above and below ground contact and blocked chemical and light-mediated signals plants normally exchange. The experimental units consisted of a group of petri dishes, each one containing chilli seeds, which were sandwiched between layers of 2 mm thick felt to retain moisture and ensure darkness. Petri dishes were arranged in a circle around a sealed central cylindrical box (as per Figure 1a). The seal at the base of the central cylindrical box, which either contained an adult plant or was left empty (control), ensured that seeds were chemically isolated from these adult plants (see Text S1 & Figure S1 for details on the Chemical testing of the experimental unit). All seeds and adult plants within a replicate unit were then housed within 2 different sized square boxes (44×44×50 cm and 32×32×45 cm respectively), one inside the other, with the air in between the two boxes removed using a pump to create a vacuum and thus avoid interference between adjacent experimental units at any time (Figure 1b). Each day, all experimental units were randomly re-interspersed throughout the growth room to avoid any potential artefacts due to their position in the room (e.g. light quantity and quality). Similarly, each day individual petri dishes within each unit were randomly re-arranged in the circular configuration around the central box to avoid any potential confounding effects of their position within the experimental unit. The temperature within the boxes was recorded over a period of 22 consecutive days to ensure that any difference in seed germination or growth measured between treatments was not due to differences in the temperature inside the boxes caused by the presence or absence of adult plants (see Figure S2). All treatments were exposed to identical nutrients, temperature and 12 h light:12 h dark cycle conditions.

#### (a) Heterospecific neighbor experiment

In August 2010, a total of 2,400 chilli seeds were randomly apportioned among 15 experimental units that were randomly allocated to 5 treatments, each replicated 3 times and kept randomly interspersed throughout the CER. Each experimental unit consisted of a group of 8 petri dishes, each of which contained 20 seeds. Petri dishes were arranged at c.10 cm from each other and in a circle around the sealed central cylindrical box. The central cylindrical box either contained an adult fennel plant or was left empty (control). All seeds and adult plants within a replicate unit were housed within the 2 different sized square boxes as described above. Treatments included: F open (i.e. adult fennel positioned in the center of the experimental unit but not enclosed in the sealed cylindrical box to allow communication via both airborne chemical and light wavelength signals); F closed (i.e. an adult fennel positioned in the center of the experimental unit and sealed in the cylindrical box to block all communication via both airborne chemical and some light wavelength signals); F masked (i.e. an adult fennel positioned in the center of the experimental unit, sealed in the cylindrical box covered in black plastic to block communication via both airborne chemical and all light wavelength signals); Control (i.e. no plant in the central cylindrical box), and Control masked (i.e. no plant in the central cylindrical box, which was covered in black plastic to account for any effects of the color of this shield itself). These treatments were carefully chosen to allow us to look at several specific independent contrasts based on four a-priori hypothesized fixed effects of particular interest: an Atmospheric Effect, a Light Effect, a Masking Effect, and an ‘Other Effect’, as explained in detail in the statistical analysis section below.

#### (b) Follow-up germination experiment

In May 2011, we repeated the experiment and increased our sample size to a total of 3,600 chilli seeds which were randomly apportioned among 3 of the original 5 treatments (i.e. F open, F masked and Control masked), each replicated 4 times. These treatments were carefully chosen to allow us to look very carefully at the ‘Other Effect’, as explained in the statistical analysis section below. The experimental units consisted of a group of 12 petri dishes, each one containing 25 seeds. In both years, seeds were inspected and watered every 24 hrs. To avoid any potential atmospheric exchange of volatiles that could have interfered with our measurements, each experimental unit was transferred one at a time to a separate room where the 2 external square boxes were opened; all petri dishes were then removed and inspected, while the rest of the unit (including the base and the central cylindrical box was taken outdoors and opened. This procedure was conducted to aerate the fennel plants sealed in the box, but was done for all units. Germination rates in each treatment were monitored and recorded every other day until 90% germination rates had been reached in at least one of the treatments (unless the number of germinating seeds reached an asymptote beforehand).

#### (c) Neighbor identity experiment

Ninety-six chilli seeds were randomly apportioned among 3 treatments (Chilli, Fennel, and Control), each replicated 4 times. The experimental units consisted of a group of 8 seeds, individually sowed into small pots (3×3×7 cm) filled with coco fiber substrate (Organic Nutrifield Coco), which were positioned c.10 cm from each other in a circle around the sealed central cylindrical box as per above. All seeds and plants within a replicate unit were then housed within the boxes and the entire unit was maintained in isolation from adjacent ones as described above. The coco fiber substrate was kept moist by watering and fertilizing every 4th day. Seeds were maintained individually in pots throughout the experiment and allowed to grow in isolation from siblings to avoid the confounding effects of root interactions and unequal acquisition of resources such as water, nutrients and light on germination and growth. On the 7th day after sowing, all seeds were inspected by lightly brushing away the top coco fibers to expose the seed using a fine paintbrush. Germination rates in each treatment were recorded and monitored for the initial 20 d of the experiment after which the number of germinating seeds reached an asymptote. Emergence rates, maximum stem height (as an estimate of above-ground growth) and number of leaves were monitored and recorded over the course of the experiment with the number of branches recorded at the conclusion at 38 d. At the end of the experiment, the roots of all seedlings were carefully washed clean of all coco fibre and photographed against a scale bar. Maximum root length (as an estimate of below-ground growth) was then measured from these calibrated digital images using the image analysis programme, OPTIMAS 6.5.

#### (d) Follow-up growth experiment

In May 2011 we conducted another experiment, where a total of 3,600 chilli seeds were randomly apportioned among 3 treatments (i.e. F open, F masked and Control masked), each replicated 4 times. The experimental units consisted of a group of 12 petri dishes, each one containing 25 seeds. At 14 d post-emergence, 240 seedlings across all treatments were removed from the experimental units and their stems and roots were measured. They were then transplanted individually into small pots filled with an identical mixture (3∶1) of sterilized soil and sand, and transferred to a shared ‘fennel-free’ environment in a glasshouse. Stem height was recorded over time and maximum root length was measured at 38 d post-emergence as per above.

### Statistical Design and Analyses

#### (a) Germination data

In the 2010 experiment, very little germination had occurred at day 4 and almost all seeds had germinated at day 11, so germination data at days 6 and 8 was used for statistical analysis. All statistical analyses were carried out in R using the base package and the lme4 package [26]. A number of generalized linear mixed models (GLMM) with binomial errors (appropriate for proportion data) were fitted to these data and the resulting models were compared in terms of Akaike’s Information Criterion (AIC) and a chi-squared test (where possible). AIC values were computed for each of the candidate models and the model with the lowest AIC value was selected as the best model of the observed data in the standard way [27]. First, a full model with a fixed effect for treatment and a continuous random time effect for Petri dish nested within experimental container was fitted to the data. The random effect accounted for the possibility that seeds within a container were affected by some conditions particular to their dish and/or container, and were thus not truly independent replicates. Since both dish and container random effects were highly significant (P<0.001), we included them in subsequent models. We next compared the full model to a model with no fixed effect for treatment, as an overall test of difference between treatments. Since this was significant, we then proceeded to look at several specific independent contrasts based on the four a-priori hypothesized fixed effects of particular interest:

Atmospheric Effect: an effect caused by the presence of the plant that acts through atmospheric contact, such as volatile chemical signals, and is thus blocked by the central cylindrical box (note that this may also incorporate some light signals based on far-red light, since the barrier blocking chemical signals also blocked far-red light).

Light Effect: an effect caused by the presence of the plant that acts through light that is not blocked by the box but is blocked by the masking

Other Effect: another effect caused by the presence of the plant that acts at a distance, is not mediated by light or atmospheric contact, and is thus not blocked by the box or masking

Masking Effect: a masking effect, caused by having the masking in the container

We then assumed that these effects were involved in the 5 treatments as follows:

F open: Atmospheric Effect, Light Effect, Other Effect (no Masking Effect)

F closed: Light Effect, Other Effect (no Atmospheric Effect or Masking Effect)

F masked: Masking Effect, Other Effect (no Atmospheric Effect or Light Effect)

Control masked: Masking Effect only (no Atmospheric Effect, Light Effect, or Other Effect)

Control: None of the effects

and we thus defined binary present/absent factors for each of the four effects across the five treatments. It was then possible to test the significance of each of the four effects directly by comparing models as follows:

Masking Effect: two models with and without the Masking effect fitted to a data subset consisting of the Control masked and Control treatments

Atmospheric Effect: two models with and without the Atmospheric effect fitted to a data subset consisting of the F open and F closed treatments

Other Effect: two models with and without the Other effect fitted to a data subset consisting of the Control masked and F masked treatments

Light Effect: two models with and without the Light effect fitted to a data subset consisting of all treatments except the F open treatment. (In this case we needed to account for the effects of Other and Masking as well, so we included both these effects in both the models).

We note that this method of specifying a-priori effects of interest and then specifying independent (orthogonal) contrasts to test these effects is generally considered more rigorous and powerful than using post-hoc pair-wise comparisons [28]. The comparisons used may seem confusing, but this degree of complexity was necessary. For example, since the masking is required to stop the transmission of all light, it is impossible to have a simple treatment-control combination that directly tests for the effect of light signaling without a masking effect. However, the design with the five treatments used in the first germination experiment allowed us to test for the separate effect of masking, which in turn allowed us to test for the effect of light signaling while accounting for the masking effect. We believe the approach used is the only way to test separately for the effects in which we were interested. In any case, it certainly allowed us to test for the ‘Other Effect’ which was the main focus of the study. In addition to these four specific a-priori hypothesized effects, we also tested for a significant difference between the Control treatment and the open fennel (F open) treatment, and between the Control and Control masked treatments (see SI for tabular presentation of tested effects; Table S1).

The analysis for the 2011 *Follow-up germination experiment* was similar to that described above, using binomial GLMMs, except there were only three treatments. Random effects for Petri dish and box were again significant, so included in all subsequent models. Models with and without a treatment effect were compared to test for overall significance of any treatment effect, and then a contrast was made between the Control masked and F Masked treatments to test specifically for an ‘other’ effect.

#### (b) Growth data

Each measured variable was analyzed separately using GLMMs. For the 2010 experiment, the number of branches at 38 days was modeled with a Poisson GLMM (appropriate for count data) with a fixed effect for treatment and a categorical random effect for plant nested within experimental container. The number of seeds germinating over time and the number of seeds emerging over time were both modeled with a binomial GLMM with fixed effects for treatment, time and an interaction between them, and a continuous time random effect for plant nested within experimental container. The number of leaves on the plant over time was modeled with a Poisson GLMM with fixed effects for treatment, time and an interaction between them, and a continuous time random effect for plant nested within experimental container. For germination, emergence and leaf number, all times were included in a single analysis. The height of the plant over time was modeled with a Gaussian GLMM (appropriate for continuous data with approximately normally distributed residuals) with fixed effects for treatment, time and an interaction between them, and a continuous time random effect for plant nested within experimental container. Only plants that had emerged by day 14 were included in this height analysis. Furthermore, since initial data exploration indicated that heights diverged over time with maximum divergence at day 29, one analysis was done with all times included, a second analysis with the last four measurement times (days 25, 29, 34 and 38) together, and a third analysis with just the day 29 measurement. The third analysis had no time effect included in the model of course. Stepwise model simplification based on AIC values was used to test whether the random effect for experimental container and the fixed effect for treatment should be included in the model. Where treatment was significant, we made specific contrasts by defining a new factor based on grouping two of the treatments at a time, refitting the model, and comparing the refitted model to the original model.

For the 2011 *Follow-up growth experiment* we conducted a similar analysis using Gaussian GLMMs with fixed effects for treatment and a random effect for experimental container, but there were only 3 treatments. The dependent variables considered were the final maximum root length and the total above-ground growth. As no significant treatment effects were found, no further comparisons were conducted.

**Table 1:** Differences due to treatment overall and to 4 specific effects.

Effect	P-values	increase/decrease	AICs with/without effect
Treatment overall	<0.0001*	–	724** vs 751
Masking	0.0289 *	decrease	335** vs 338
Atmospheric	0.0049 *	decrease	279** vs 285
Light	0.5257	–	532 vs 530**
Other	0.0086 *	increase	243** vs 248

*Notes:* Shown are P-values for significance of effect obtained from a chi-squared test of deviance, AIC values for models with and without the effect, and, if significant, whether specific effects increased or decreased germination at days 6 and 8. Significance at P<0.05 indicated by * and model with lower AIC, which is the preferred model, is indicated by **.

## Results

**Figure 2:**
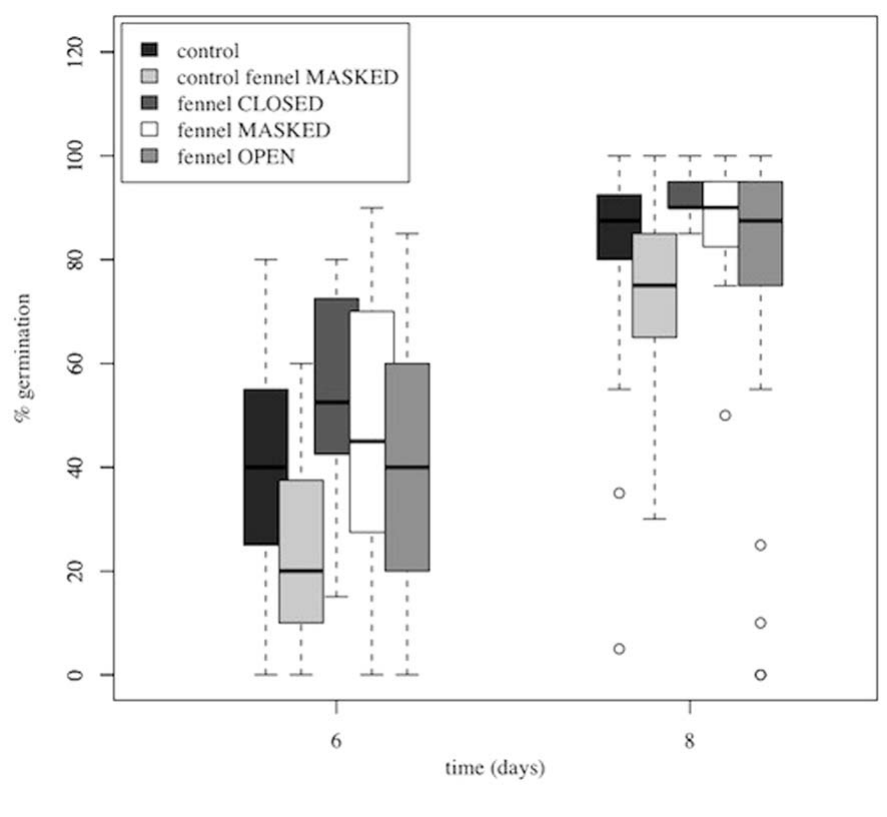
Germination of chilli seeds is affected by the mere presence of an adult fennel plant. Because very little germination had occurred at day 4 and almost all seeds had germinated at day 11, germination data at days 6 and 8 are presented and used for statistical analysis. The median, inter-quartile range and range are represented by the middle bar, the top and bottom of box and the whiskers respectively. Outliers laying more than 3 times the inter-quartile range from the median are represented by the small circles. * n is total number of seeds as appropriate for binomial analysis.

In our first germination experiment, we found a significant overall effect due to Treatment, and specific Atmospheric, Masking and Other effects (GLMM, P<0.0001; Table 1). The difference between the Control and the F Open treatments were not significant (Figure 2). Nonetheless, the percentage of seed germination over time was higher in the 3 treatments where the fennel was present than in the two controls (ΔAIC = 2.9; P = 0.027) and, seeds germinated significantly faster when fennel was present, even when all known signals from the fennel were blocked (Control masked vs F masked; ΔAIC = 4.9; P = 0.009). The masking effect was confirmed by a significant difference between the Control and Control masked treatments (ΔAIC = 2.8; P = 0.029). When this experiment was repeated in the following year with an increased sample size (the 2011 *Follow-up germination experiment*), we found again a strongly significant positive Other effect (F masked > Control masked; P = 0.005; Figure S3).

**Figure 3:**
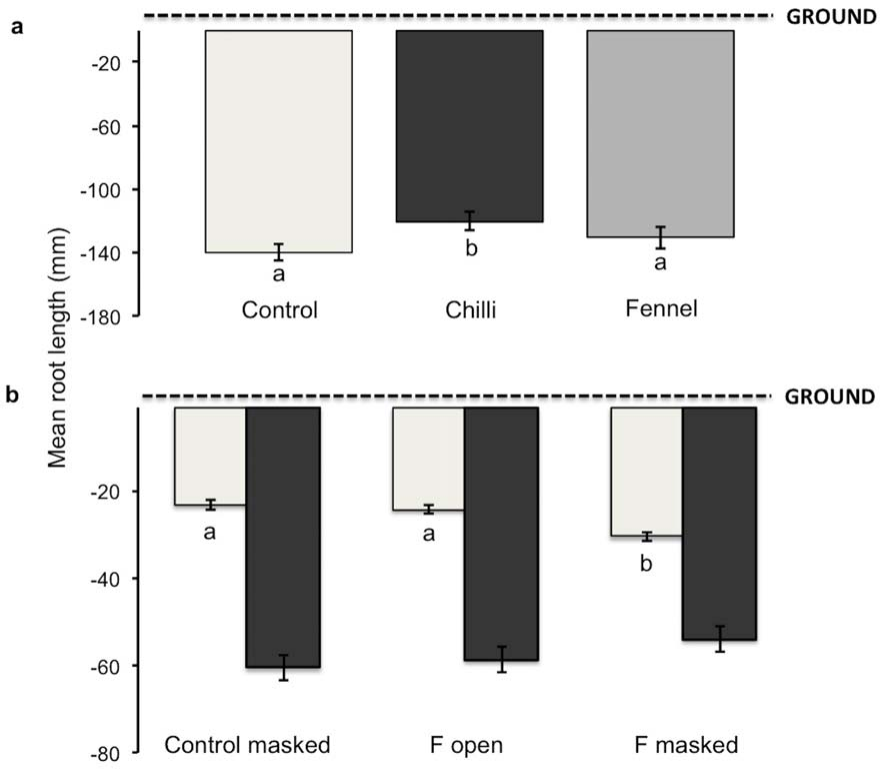
Mean final root size of chilli seedlings is affected by the presence and identity of their adult neighbours. (**a**) Overall, maximum root length differed significantly depending on the neighbouring plant present in the sealed central box (n = 32 per treatment). Seedlings growing next to adult chilli plants had significantly shorter roots than those in the empty control or growing with the fennel (P = 0.015). (**b**) The presence of a neighbouring fennel during germination and emergence caused an increase in early root development of chilli seedlings when the communication channels are blocked, but not when unblocked (light grey bars) (F masked > F open and Control masked; P = 0.027; n = 80 per treatment). Differences disappeared when seedlings were allowed to grow away from a fennel plant (dark grey bars) (P = 0.94; n = 80 per treatment). Error bars indicate standard errors.

**Figure 4:**
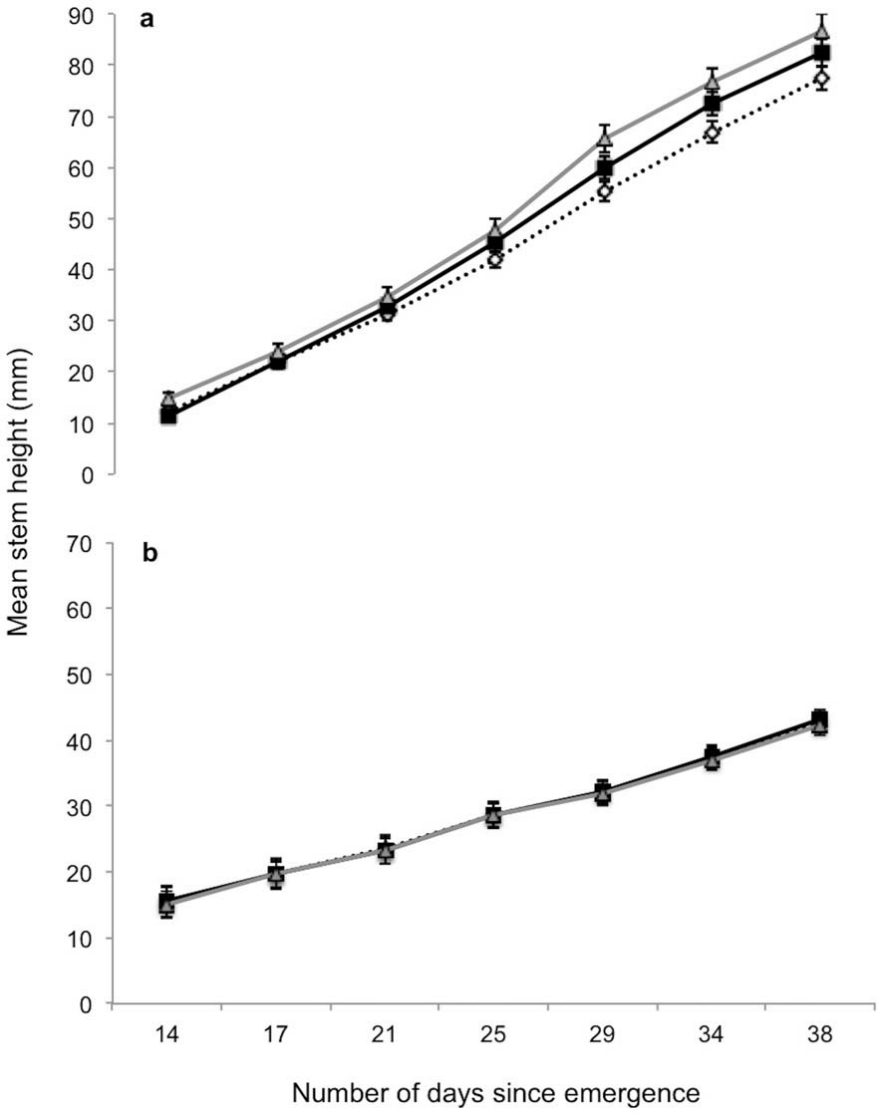
Early growth of chilli seedlings depends on the presence and identity of their neighbour. (**a**) Seedlings growing next to a fennel (grey solid line and triangles) are marginally significantly taller than those growing next to an adult chilli plant (black solid line and squares; Pair-wise contrasts, P = 0.07) and significantly taller than seedlings in the empty control (black dotted line and white diamonds; Pair-wise contrasts, P = 0.01). The observed differences in above-ground growth among treatments (adult fennel plant, grey solid line and triangles; adult chilli plant, black solid line and squares; empty control, black dotted line and white diamonds) are amplified over time. Only plants that emerged by day 14 are included in these analyses (n = 32 per treatment). Error bars indicate standard errors. (**b**) Growth differences disappear when seedlings are allowed to grow in the absence of any adult plant after emergence (n = 80 per treatment). Error bars indicate standard errors.

Additionally, chilli seedlings growing adjacent to an adult conspecific allocated significantly less to their roots than did seedlings growing adjacent to an adult fennel or in the control (ΔAIC = 2.6; P = 0.047; Figure 3a). We did not find that the number of leaves or branches varied across treatments (P = 0.88 leaves; P = 0.82 branches). However, seedling height did differ significantly (ΔAIC = 2.9; P = 0.04). Generally, we found that such differences among treatments were magnified over the course of the experiment (Figure 4a). Interestingly in the *Follow-up growth experiment*, where seeds were initially germinated in the presence of a neighbouring fennel (i.e. F open and F masked) and then allowed to grow away from it, there were no significant differences in final growth (P = 0.83 roots; P = 0.53 shoots; Figure 3b and 4b).

## Discussion

By selectively blocking above- and below-ground contact, chemical and light-mediated signals, our study revealed the existence of uncharted communication channels used by seeds and seedling to sense neighbors and identify relatives. Most intriguingly, we found that chilli seeds developing in the F masked treatment (i.e. plant present but all communication via direct contact and airborne chemical and light wavelength signals was blocked) germinated significantly faster than those in the Control masked treatment, which contained no fennel plant and was designed merely to account for the ‘masking’ effect of the black plastic shield (i.e. positive ‘other’ effect). This effect was observed in both the original and the follow-up germination experiments. However, seeds germination was accelerated in treatments where fennel was present but its signals were partially (F closed) or totally blocked (F masked treatment) than in the treatment where fennel was present and its signals not blocked, suggesting that light or volatile chemical signals from fennel plants must be hindering the chilli seeds' germination rates (i.e. negative ‘atmospheric’ effect). We concluded that the lack of a significant difference between germination in the Control and F Open treatments must thus be a result of two different signals cancelling out, a negative effect due to light and/or chemical signals and a positive effect due to something else. Because our understanding of the interplay between different signalling pathways is generally still rudimentary, the full biological meaning of the interactive effect observed here remains unclear. Interestingly, the study of how plants integrate multiple interacting signals and for example, how plants might integrate light and the signalling pathways of hormones such as jasmonate to modify their growth and development, while responding to encroaching neighbours, has become an increasingly key topic of recent research (reviewed by [29]). In this context, our results further confirm the complex nature of interactions between different signalling pathways and call for a better understanding of these processes.

The fact that chilli seeds growing next to a masked fennel exhibited significantly faster germination rates than conspecifics growing in the masked control indicated that these seeds were somehow able to discriminate the presence of a plant even when it was fully masked and isolated in a box. This finding is particularly interesting because it emphasises the importance of biotic cues regarding the presence and identity of neighbours as an influence on germination timing. Previous studies have shown that seeds adaptively use germination cues and accelerate germination in very competitive environments and/or where seeds that do not germinate quickly may be prevented from germinating at all [e.g. [30]–[31]. A good explanation for the observed results is that accelerated germination of chilli seeds is triggered by a non-chemical signal. The acceleration in germination could be a strategy to counteract allopathic inhibition of germination at this early stage and also possible future allopathic inhibition of growth. However, in normal situations germination of chilli seeds is also inhibited by an allopathic chemical signal from the fennel plants, which offsets the acceleration in germination triggered by the separate non-chemical signal. When the allopathic chemical signal is blocked, the seeds still identify the presence of the fennel through the non-chemical signal and respond accordingly with accelerated germination that is not offset by allopathic inhibition. Another possible explanation is that both responses are adaptive. The non-chemical signal could be a more general signal indicating the presence of a possible competitor and thus triggering faster germination, while the chemical signal could be more specific, indicating that this particular species has particular characteristics that make slower germination more beneficial.

Because germination rates have lifelong fitness consequences [32], selection should clearly favour mechanisms allowing a plant to detect its neighbours and hence its forthcoming competitive environment and regulate its developmental responses accordingly at the very onset of its life (i.e. seed stage). Indeed, it is known that germination is triggered by environmental cues [17], [33] and plants have developed numerous ways to assess the most favourable time for germination based on the quality of their surrounding environment, including the density of neighbouring seeds, seedlings and adult plants [34]–[35]. The novelty of our findings here is the evidence for the existence of an as yet unidentified mechanism allowing seeds to sense their adult neighbourhood, in this case a fennel, prior to emergence without direct contact between them, through light or chemical signals, either above or below ground. We note that our results show differences in the speed of germination, not total germination, because differences in germination had disappeared by the 11^th^ day.

In addition to the positive ‘other’ effect, our results also showed evidence for a negative atmospheric effect and a negative masking effect. Possible explanations for the negative atmospheric effect and the way it interacted with the ‘other’ signal are provided above. The result that masking itself inhibited germination is also very interesting, but is perhaps more easily explained as an effect of differences in the amount of type of light being reflected. Fittingly, our results on the germination of chilli seeds in the ‘natural’ presence of a fennel plant (i.e. without any restriction in communications) compared to the other treatments that each restrict a different subset of the potential signalling mechanisms suggest that germination is informed by the integration of multiple interacting stimuli signalling the current growth environment (e.g. who your neighbours are) and possibly mediating the most cost-effective germination response (e.g. whether suppression or acceleration in germination rates is required in the presence of a fennel plant). However, the design of the current study was focussed on testing for the alternative means of communication, rather than the many other effects involved. We must note again that ‘atmospheric’ effect may also include effects of some wavelengths of light signals (see Methods), and the fact that ‘light’ effect was found to be non-significant may be because important light effects were actually accounted for in the ‘atmospheric’ effect. It would be interesting in future work to design experiments that more precisely target and disentangle the interactive effects of the different light signals, the chemical signals and also the effects of the masking and the cylinder itself, through adding an extra control without a cylinder for example.

When we further explored this unexpected effect by testing whether different neighbouring species affected chilli germination rates and also subsequent growth when we blocked all known communication channels, we found that seedlings allocated energy to their stem and root systems differently depending on the identity of the neighbour. For instance, chilli seedlings growing adjacent to an adult conspecific allocated significantly less to their roots than did seedlings growing adjacent to an adult fennel or in the control. This finding is consistent with the idea that recognising a neighbour as kin becomes advantageous to prevent costly competitive behaviour toward relatives (i.e. kin selection; [34], [36]). Clearly, roots represent a complex underground communication system for plants and much information about their surroundings and neighbours is transmitted via root interactions. Interestingly, our results demonstrate that chilli plants exhibit responses consistent with those described above, but in the absence of a physiological connection by roots with the neighbouring plants. These findings are similar to those recently presented by Karban and Shiojiri [37], who demonstrated that the sagebrush *Artemisia tridentata* was able to discriminate self from non-self in the absence of physical contact. Although the mechanisms of recognition were not known at the time, the authors suggested that volatile determinants were the likely candidate (see also [38]). Because our experimental setup ensured that no volatile chemicals from any of the adult plants could interfere with seedling growth, we are able to demonstrate that both physical connection by roots and physical interaction via volatile chemicals with the neighbouring plants are not indispensable requirements for the mechanisms of recognition to occur.

Apart from plasticity in root allocation, a suite of above-ground traits such as number of branches, plant height and/or stem elongation are known as candidate traits for examining competitive responses of plants to the presence of other neighbouring plants and better understanding their physiological ecology of resource acquisition and allocation. In this study, we observed no changes in the number of leaves or branches across treatments, but significant differences in seedling height. For example, chilli seedlings were consistently taller when growing next to an adult fennel than an adult chilli plant, despite there being a constant amount of space and nutrients per individual across all treatments and for the entire duration of the experiment. Moreover, seedlings were taller when they shared their space with another plant than in the control treatment (i.e. no plant). While stem elongation responses differ among species and within populations [39]–[41], extension of stem height is well-known to be a competitive response to neighbours, when their presence affects the quality of light by reducing the red to far red ratio (R:FR) of incident light (i.e. shade avoidance syndrome; [42]). For example, Collins and Wein [41] showed that competition with neighbours resulted in stem elongation in the arrow tearthumb, *Polygonum sagittatium*, allowing it to tower over neighbouring plants and therefore mediating such a shade avoidance response. While it is interesting that seedling responses to neighbour presence in our experiment were consistent with this shade avoidance syndrome, it is the fact that they did so in the absence of R:FR light (or any chemical) signals from the putative competitor that is remarkable.

Additionally, the more pronounced overall elongation response that we observed in the *Neighbor identity experiment* when chilli seedlings grew with a stranger (i.e. fennel) rather than a relative (i.e. chilli), further demonstrate that these plants can recognise their neighbours, and compete more strongly with strangers and potentially reduce interfere when growing next to relatives (see [14]). While we cannot completely exclude the possibility that neighbour recognition by chilli seedlings was facilitated by changes in (visible) light, our results do not support the involvement of recognition based on changes in R:FR light, because our experimental units were purposely opaque to infrared wavelengths. Moreover, our follow-up growth experiment further convinced us that the observed growth effects were indeed due to the presence of a neighbour in the experimental units during growth.

In conclusion, we have demonstrated that young chilli plants are able to sense their neighbours from as early as the seed stage. Furthermore as seeds grow into seedlings, they are able to discriminate among neighbouring species and modify their growth patterns accordingly, without necessarily relying on known determinants, such as volatile chemicals, direct physical contact or changes in infrared light wavelengths. Together, these findings beg the question: which other determinants may be operating to facilitate recognition? We do not know yet what mechanism(s) may be mediating such responses. There is a large and convincing body of experimental evidence demonstrating that plants are highly sensitive to the Earth’s geomagnetic field (GMF; i.e. gravity), which is a natural and permanent component of their environment [43]. Perhaps they can also perceive other magnetic fields of low intensity (i.e. weak magnetic field, WMF), particularly during seed germination. If so, can plants detect the magnetic fields generated by other plants? And how strong are plant-generated magnetic fields? Because the strength of a magnetic field decreases rapidly as the distance from its source increases, information of magnetic nature would be useful for close-range communication only, where the distance of a receiving plant from an emitting plant would be of fundamental importance. In this context, this information would be particularly valuable to seeds and seedlings monitoring their immediate surroundings to identify potentially unfavourable neighbours. Indeed, previous research on the effects of magnetic fields on seeds has reported both inhibition and stimulation of the germination process depending on the study species, the intensity of the field applied and the duration of exposure (reviewed in [43]). If plants are characterised by species-specific fields with varying intensities, this could be a possible explanation for the apparently conflicting results. We believe that the hypothesis that magnetic fields may be used to convey information at close-range is a testable option worth exploring. Additionally, sound may be another modality by which plants exchange information. Decades of scientific research has measured and described sound waves produced by plants as well as the effects of sound on plants such as changes in germination and growth rates as well as physiological responses (reviewed in [44]). Moreover, both emission and detection of sound may have adaptive value in plants and while we still don’t know how sound is perceived in that we are yet to identify receptor mechanisms and study their function, we have clear evidence about plants’ ability of detecting vibrations and exhibiting a selective sensitivity on the basis of which they modify their behavior (e.g. root growth; [45]). This research offers a particularly exciting opportunity to study and understand plant communication and opens a stimulating debate on our view of these organisms.

## Supporting Information

Figure S1 **Chemical testing of the experimental unit.** Mean concentration of volatile anethole detected in different compartment of the experimental unit after 24 hr exposure. Volatile anethole was easily detectable and at high levels when the SPME fiber was sealed inside the central cylindrical box. However when the fiber was placed within the outer compartment of the experimental unit while the volatile anethole was sealed within central cylindrical box, GC/MS readings were not detectably different from the background readings performed with an empty box and in the absence of anethole (One-way ANOVA, F2, 6 = 369.95, P<0.0001). Error bars indicate 95% CI (n = 3 per treatment).
(DOCX)

Figure S2 **Temperature profile within the experimental units.** Mean temperature profile recorded inside the experimental unit over 24 hrs. The presence (i.e. F open, black dotted line; F masked, black solid line) or absence of an adult plant within the box (i.e. Control masked; grey solid line) had no effect on the temperature profiles seeds would experience within the box (Repeated-measure ANOVA, F46, 115 = 1.10, P = 0.33). Error bars indicate 95% CI (n = 5 per treatment). Temperature data were collected using an U12-011 - HOBO® Temperature/RH Data Logger.
(DOCX)

Figure S3 **Germination of chilli seeds across treatments.** Germination of chilli seeds is affected by the mere presence of an adult fennel plant. The percentage of seed germination over time is higher when the fennel is present but all known signals are blocked (grey boxes). The median, inter-quartile range and range are represented by the middle bar, the top and bottom of box and the whiskers respectively. Outliers laying more than 3 times the inter-quartile range from the median are represented by the small circles.
(DOCX)

Table S1 **Tested effects in each treatment.** The number 1 indicates that an effect was operating in a particular treatment, while the number 0 indicate it was not operating.
(DOCX)

Text S1 **Chemical testing of the experimental unit.** Details on method validation to determine whether the experimental unit was volatile-proof.
(DOCX)

## References

[1] Scott-Phillips TC (2007). Defining biological communication..

[2] Carazo P, Font E (2010). Putting information back into biological communication..

[3] Baldwin IT, Schultz JC (1983). Rapid changes in tree leaf chemistry induced by damage: evidence for communication between plants..

[4] Dicke M, Agrawal AA, Bruin J (2003). Plants talk, but are they deaf?.

[5] Baluska F, Mancuso S, Volkmann D (2005). Communication in plants, 1^st^ edn..

[6] Karban R 2008 Plant behavior, Ecol Lett 11 communication, (doi: 10.1111/j 727–739 (1461–0248).

[7] Smith H (1995). Physiological and ecological function within the phytochrome family..

[8] Gersani M, Brown JS, O’Brien EE, Maina GM, Abramsky Z (2001). Tragedy of the commons as a result of root competition..

[9] Gruntman M, Novoplansky A (2004). Physiologically mediated self/non-self discrimination in roots..

[10] Murphy GP, Dudley SA (2007). Above- and below-ground competition cues elicit independent responses..

[11] Trewavas A (2003). Aspects of plant intelligence..

[12] Baluska F, Mancuso S (2007). Plant neurobiology as a paradigm shift not only in the plant sciences.. Plant Signal Behav.

[13] Dudley SA, File AL (2007). Kin recognition in an annual plant..

[14] Murphy GP, Dudley SA (2009). Kin recognition: competition and cooperation in *Impatiens* (Balsaminaceae)..

[15] Heil M, Karban R (2010). Explaining evolution of plant communication by airborne signals..

[16] Telewski F (2006). A unified hypothesis of mechanoperception in plants.. Am J Bot.

[17] Smith H (2000). Phytochromes and light signal perception by plants–an emerging synthesis.. Nature.

[18] Pare PW, Tumlinson JH (1999). Plant volatiles as a defense against insect herbivores.. Plant Physiol.

[19] Karban R, Baldwin IT, Baxter KJ, Laue G, Felton GW (2000). Communication between plants: induced resistance in wild tobacco plants following clipping of neighboring sagebrush..

[20] Heil M, Ton J (2008). Long-distance signalling in plant defence..

[21] Ballaré CL, Scopel AL (1997). Phytochrome signalling in plant canopies: testing its population-level implications with photoreceptor mutants of *Arabidopsis*.. Funct Ecol.

[22] Herms DA, Mattson WJ (1992). The dilemma of plants: to grow or to defend.. Quart Rev Biol.

[23] Perry L, Dickau R, Zarrillo S, Holst I, Pearsall DM (2007). Starch fossils and the domestication and dispersal of chilli peppers..

[24] Pickergill B (1971). Relationships between weedy and cultivated forms in some species of chilli peppers (Genus *Capsicum*).. Evolution.

[25] Gregg RB (1943). Companion and Protective Plants.. Bio-Dynamics.

[26] Bates D, Maechler M, Bolker B (2011). http://CRAN.R-project.org/package=lme4.

[27] Burnham KP, Anderson DR (2002). Model selection and multimodal inference: a practical information-theoretic approach, 2^nd^ edn..

[28] Sokal RR, Rohlf FJ (2012). Biometry: the principles and practice of statistics in biological research, 4^th^ edn..

[29] Kazan K, Manners JM (2011). The interplay between light and jasmonate signalling during defence and development.. J Exp Bot.

[30] Tilebörger K, Prasse R (2009). Do seeds sense each other? Testing for density-dependent germination in desert perennial plants.. Oikos.

[31] Orrock JL, Christopher CC (2010). Density of intraspecific competitors determines the occurrence and benefits of accelerated germination.. Am J Bot.

[32] Verdú M, Traveset A (2005). Early emergence enhances plant fitness: a phylogenetically controlled meta-analysis.. Ecology.

[33] Venable DL, Lawlor L (1980). Delayed germination and dispersal in desert annuals: escape in space and time..

[34] Waldman B (1988). The ecology of kin recognition.. Ann Rev Ecol Syst.

[35] Falik O, Reides P, Gersani M, Novoplansky A (2003). Self/non-self discrimination in roots..

[36] Kelly JK (1996). Kin selection in the annual plant *Impatiens capensis*.. Am Nat.

[37] Karban R, Shiojiri K (2009). Self-recognition affects plant communication and defense..

[38] Karban R, Shiojiri K, Ishizaki S (2010). An air-transfer experiment confirms the role of volatile cues in communication between plants..

[39] Geber MA (1989). Interplay of morphology and development on size inequality: a *Polygonum* greenhouse study.. Ecol Monogr.

[40] Ballaré CL, Scopel AL, Sanchez RA (1991). On the opportunity cost of the photosynthate invested in stem elongation reactions mediated by phytochrome.. Oecologia.

[41] Collins B, Wein G (2000). Stem elongation response to neighbour shade in sprawling and upright *Polygonum* species..

[42] Aphalo PJ, Ballaré CL, Scopel AL (1999). Plant-plant signalling, the shade-avoidance response and competition..

[43] Belyavskaya NA (2004). Biological effects due to weak magnetic field on plants..

[44] Gagliano M (*in review*) Green symphonies: a call for studies on sound communication in plants.

[45] Gagliano M, Mancuso S, Robert D Towards understanding plant bioacoustics..

