# Supplementary material for: Out of Sight but Not out of Mind: Alternative Means of Communication in Plants
Source: PLoS One. 2012 May 22;7(5):e37382. doi: 10.1371/journal.pone.0037382 (PMC3358309; doi:10.1371/journal.pone.0037382)
Supplement: Figure S1 — Chemical testing of the experimental unit. Mean concentration of volatile anethole detected in different compartment of the experimental unit after 24 hr exposure. Volatile anethole was easily detectable and at high levels when the SPME fiber was sealed inside the central cylindrical box. However when the fiber was placed within the outer compartment of the experimental unit while the volatile anethole was sealed within central cylindrical box, GC/MS readings were not detectably different from the background readings performed with an empty box and in the absence of anethole (One-way ANOVA, F2, 6 = 369.95, P<0.0001). Error bars indicate 95% CI (n = 3 per treatment). (DOCX) [file pone.0037382.s001.docx]

**
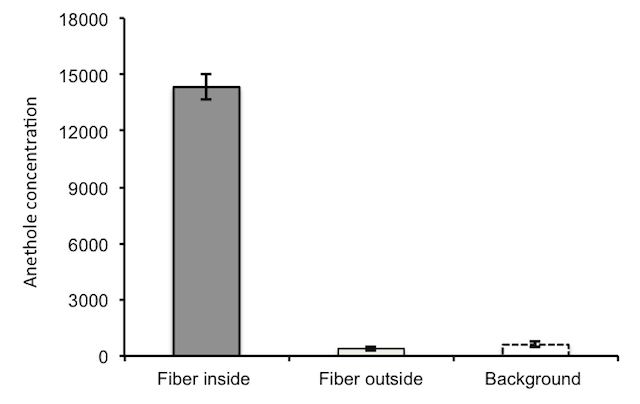
**

**Figure S1.** **Chemical testing of the experimental unit.** Mean concentration of volatile anethole detected in different compartment of the experimental unit after 24hr exposure. Volatile anethole was easily detectable and at high levels when the SPME fiber was sealed inside the central cylindrical box. However when the fiber was placed within the outer compartment of the experimental unit while the volatile anethole was sealed within central cylindrical box, GC/MS readings were not detectably different from the background readings performed with an empty box and in the absence of anethole (One-way ANOVA, F_2, 6_ = 369.95, P < 0.0001). Error bars indicate 95% CI (n = 3 per treatment).
