# Supplementary material for: Out of Sight but Not out of Mind: Alternative Means of Communication in Plants
Source: PLoS One. 2012 May 22;7(5):e37382. doi: 10.1371/journal.pone.0037382 (PMC3358309; doi:10.1371/journal.pone.0037382)
Supplement: Figure S2 — Temperature profile within the experimental units. Mean temperature profile recorded inside the experimental unit over 24 hrs. The presence (i.e. F open, black dotted line; F masked, black solid line) or absence of an adult plant within the box (i.e. Control masked; grey solid line) had no effect on the temperature profiles seeds would experience within the box (Repeated-measure ANOVA, F46, 115 = 1.10, P = 0.33). Error bars indicate 95% CI (n = 5 per treatment). Temperature data were collected using an U12-011 - HOBO® Temperature/RH Data Logger. (DOCX) [file pone.0037382.s002.docx]

**
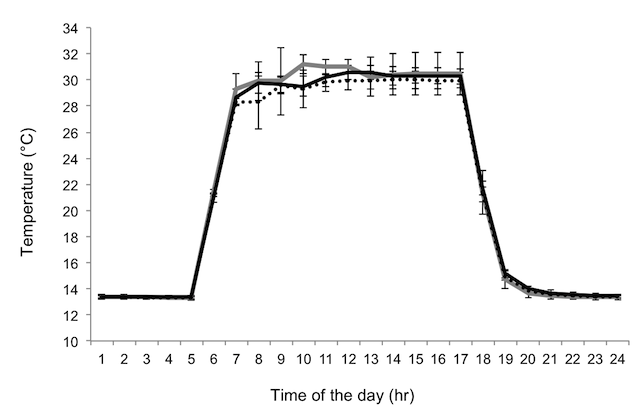
**

**Figure S2.** **Temperature profile within the experimental units.** Mean temperature profile recorded inside the experimental unit over 24 hrs. The presence (i.e. F open, black dotted line; F masked, black solid line) or absence of an adult plant within the box (i.e. Control masked; grey solid line) had no effect on the temperature profiles seeds would experience within the box (Repeated-measure ANOVA, F_46, 115_ = 1.10, P = 0.33). Error bars indicate 95% CI (n = 5 per treatment). Temperature data were collected using an U12-011 - HOBO® Temperature/RH Data Logger.
