# Supplementary material for: Out of Sight but Not out of Mind: Alternative Means of Communication in Plants
Source: PLoS One. 2012 May 22;7(5):e37382. doi: 10.1371/journal.pone.0037382 (PMC3358309; doi:10.1371/journal.pone.0037382)
Supplement: Figure S3 — Germination of chilli seeds across treatments. Germination of chilli seeds is affected by the mere presence of an adult fennel plant. The percentage of seed germination over time is higher when the fennel is present but all known signals are blocked (grey boxes). The median, inter-quartile range and range are represented by the middle bar, the top and bottom of box and the whiskers respectively. Outliers laying more than 3 times the inter-quartile range from the median are represented by the small circles. (DOCX) [file pone.0037382.s003.docx]

**
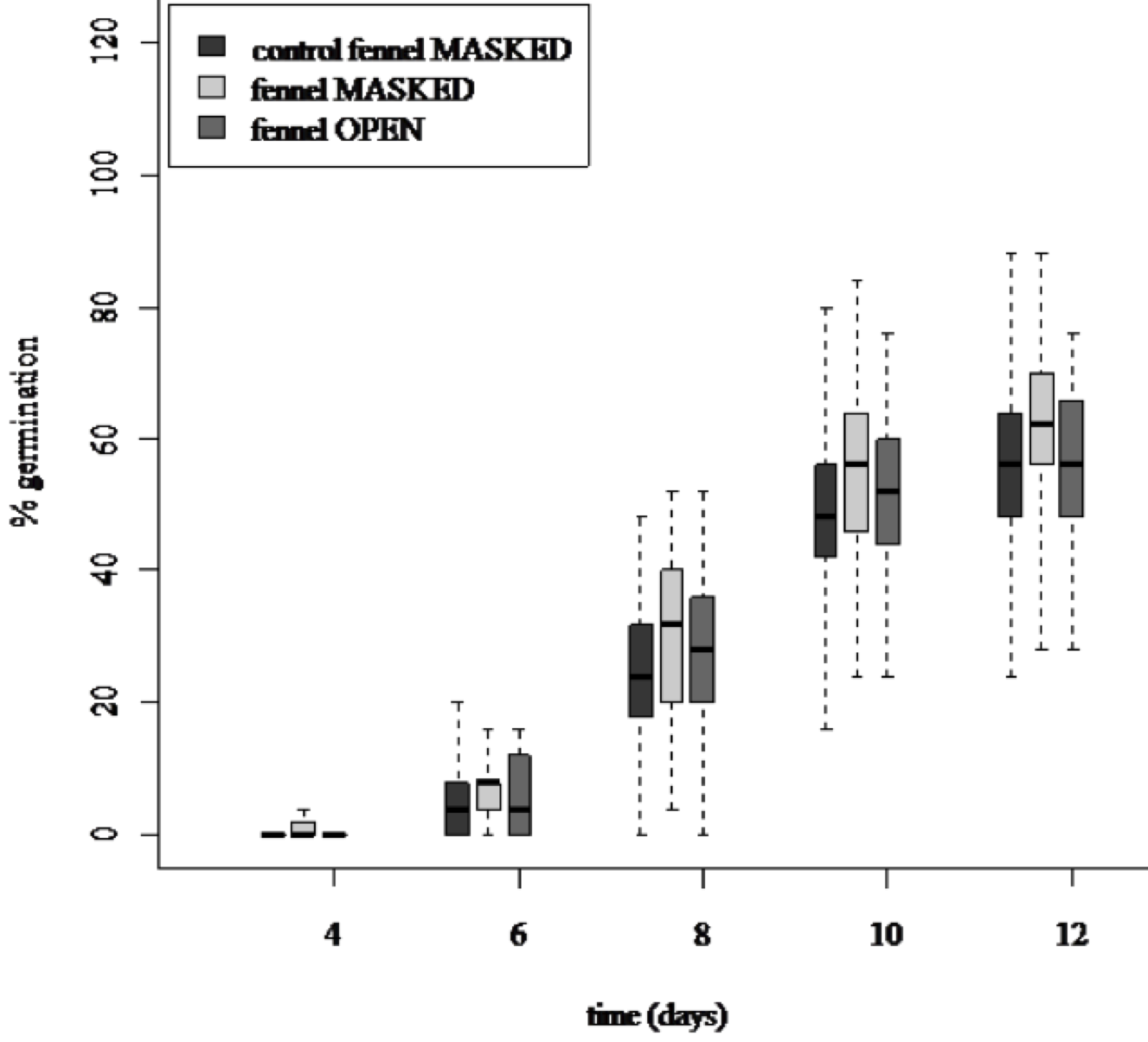
**

**Figure S3. Germination of chilli seeds across treatments.** Germination of chilli seeds is affected by the mere presence of an adult fennel plant. The percentage of seed germination over time is higher when the fennel is present but all known signals are blocked (grey boxes). The median, inter-quartile range and range are represented by the middle bar, the top and bottom of box and the whiskers respectively. Outliers laying more than 3 times the inter-quartile range from the median are represented by the small circles.
