# Supplementary material for: Out of Sight but Not out of Mind: Alternative Means of Communication in Plants
Source: PLoS One. 2012 May 22;7(5):e37382. doi: 10.1371/journal.pone.0037382 (PMC3358309; doi:10.1371/journal.pone.0037382)
Supplement: Table S1 — Tested effects in each treatment. The number 1 indicates that an effect was operating in a particular treatment, while the number 0 indicate it was not operating. (DOCX) [file pone.0037382.s004.docx]

**Table S1. Tested effects in each treatment.** The number *1* indicates that an effect was operating in a particular treatment, while the number *0* indicate it was not operating.

|  | **Light Effect** | **Atmospheric Effect** | **Masking Effect** | **Other Effect** |
| --- | --- | --- | --- | --- |
| F open | **1** | **1** | 0 | **1** |
| F closed | **1** | 0 | 0 | **1** |
| F masked | 0 | 0 | **1** | **1** |
| Control masked | 0 | 0 | **1** | 0 |
| Control | 0 | 0 | 0 | 0 |
