# Supplementary material for: Out of Sight but Not out of Mind: Alternative Means of Communication in Plants
Source: PLoS One. 2012 May 22;7(5):e37382. doi: 10.1371/journal.pone.0037382 (PMC3358309; doi:10.1371/journal.pone.0037382)
Supplement: Text S1 — Chemical testing of the experimental unit. Details on method validation to determine whether the experimental unit was volatile-proof. (DOCX) [file pone.0037382.s005.docx]

**Text S1. Chemical testing of the experimental unit.**

Anethole is a primary volatile metabolite from fennel and was chosen for method validation to determine whether the experimental unit used for these experiments was volatile-proof. Anethole of >99.5% purity was purchased from SIGMA-ALDRICH (product code 10368) and dissolved in HPLC grade ethanol. Anethole could be detected with a signal to noise ratio of >4 when 50pg (in 5µl ethanol) was evaporated into the experimental box and measured using the Solid Phase Micro Extraction (SPME) technique and GC/MS analyses. Specifically, SPME experiments were performed using a polydimethylsiloxane fibre from Supelco (product code 57362-U). Fibres were conditioned in the GC injection port at 280°C under helium flow in accordance with the manufacturer's recommendations. Extractions inside the inner cylindrical box and in the outer compartment of the experimental box were performed for 24 h. Each experiment was performed in triplicate with three separate fibres. Fibre blanks were run in between each sample run to remove carryover. Samples were analysed using an Agilent 7890A Gas Chromatograph (GC) equipped with a 5875C Mass Selective Detector (MSD) and Gerstel MPS autosampler. Helium was used as the carrier gas at a constant flow of 1 ml/min. Inlet temperature was set at 280°C. The fibre was desorbed in the inlet for 2 min, at which point the septum purge initiated; the fibre was then held in the inlet for a further 2 min. Oven temperature was initially set at 80°C for 1min, ramped at 20°C/min to 280°C, then held at this temperature for 10 min. A Varian Factor 4 capillary column (VF-5ms, 30 m x 0.25 mm, 0.25 µm plus 10m EZ-Guard) was used for separation. The MSD transfer line heater was kept at 280°C. MS quadrupole temperature was 150°C and source temperature was 230°C. The MSD was run in scan and select ion monitoring (SIM) mode simultaneously; masses from 30 - 300 amu were scanned and the major fragmentation ion of 148 amu from anethole was monitored in SIM mode. Data analysis was performed using Agilent's MSD Chem Station (2008).
